# Melphalan intra-arterial chemotherapy for choroidal melanoma chemoreduction

**DOI:** 10.1186/s40942-022-00404-1

**Published:** 2022-08-17

**Authors:** Rodrigo Jorge, Igor Coelho, Gustavo Viani, Amanda Alexia R. Vieira, Fernando Chahud, Daniel G. Abud, Zelia M. Correa

**Affiliations:** 1 Department of Ophthalmology, Ribeirão Preto Medical School, University of São Paulo, 3900, Bandeirantes Ave, SP Ribeirão Preto, 14049-900 Brazil; 2 Department of Medical Images, Hematology and Oncology, Ribeirão Preto Medical School, University of São Paulo, Ribeirão Preto, Brazil; 3 Department of Pathology, Ribeirão Preto Medical School, University of São Paulo, Ribeirão Preto, Brazil; 4 Ocular Oncology Service, Bascom Palmer Eye Institute, University of Miami, Miami, FL USA

**Keywords:** Melanoma, uveal, Chemotherapy, Intra-arterial, Melphalan, Ruthenium-106

## Abstract

**Background:**

Intra-arterial chemotherapy (IAC) has been used to treat multiple cancers including liver metastasis from uveal and cutaneous melanoma but not as primary tumor treatment. We report the compassionate use of chemoreduction with intra-arterial melphalan before ruthenium brachytherapy to salvage an eye with choroidal melanoma.

**Case presentation:**

A 61-year-old female patient complained of decreased vision and central-temporal scotoma in OS (left eye) for 1 month. Visual acuity was 20/20 in right eye (OD) and 20/125 OS. Anterior segment examination and intraocular pressure were unremarkable in both eyes, as was fundus examination of the OD. Fundus examination of OS revealed a brown, solid tumor partially obscuring the temporal optic disc margin and extending to the equatorial fundus midzone. Serous retinal detachment was present over the lesion and around it. Ultrasonography revealed a solid choroidal tumor with a largest basal diameter (LBD) of 13.0 mm and thickness of 10.4 mm. The tumor presented acoustic hollowness and a superimposing retinal detachment. After metastatic screening was negative, the patient underwent intra-arterial chemotherapy with melphalan. Three weeks later, her visual acuity was 20/200 and there was noticeable tumor regression to 11.9 mm (LBD) by 7.9 mm (thickness) allowing brachytherapy to be performed. Ten weeks after brachytherapy (13 weeks after IAC), visual acuity was HM due to biopsy-related vitreous hemorrhage (VH). Tumor dimensions were 9.9 (LBD) mm and 6.5 mm (thickness) and PPV was performed to remove VH. Six weeks after PPV (20 weeks after IAC), her visual acuity was 20/200 and further reduction of tumor dimensions was observed: largest basal diameter was 8.9 mm and thickness was 4.9 mm.

**Conclusion:**

This case illustrates the feasibility of combining induction IAC prior to ruthenium brachytherapy for large choroidal melanoma. More studies are warranted to confirm these early preliminary findings.

## Introduction

The management of choroidal melanoma has been the focus of extensive research because it is the most common primary intraocular tumor in adults [1]. Maximizing local disease control, reducing the risk of distant metastases, and avoiding enucleation are the primary treatment goals in managing this disease [2]. Currently, brachytherapy is considered the standard treatment for small and medium tumors [3]. In clinical practice, ruthenium (Ru-106) or iodine (I-125) are the most common brachytherapy sources employed for small and medium tumors. However, there are severe complications associated with the utilization of these radioactive sources and this use is limited for large choroidal melanomas (tumors > 10 mm in thickness and > 22 mm in diameter) [3]. The limitations of brachytherapy for such tumors stems from its inability to deliver an adequate therapeutic dose due to the need to deliver high doses to the sclera that translates into poor outcomes and severe complications. In this context, in the last decades, other treatment options such as external beam radiotherapy (EBRT), radiosurgery (Gamma Knife or linear accelerator), and proton therapy has been investigated [4].

Although gamma knife radiosurgery and proton therapy are effective treatments with reliable tumor control rates, they are associated with poor visual outcomes, inadequate globe retention, and their use is reportedly limited to the public health system of low- or middle-income countries [5, 6]. EBRT carries the risk of significant side effects related to a broader radiation field and increased risk of complications ocular and periocular structures such as eyelids, sclera, lens, and retina [7]. Finally, enucleation is an effective management for very large tumors and usually a last resource.

Considering the above-mentioned treatment limitations for large choroidal melanomas, our group explored an alternative approach using intra-arterial with melphalan for choroidal melanoma chemoreduction to reduce tumor thickness and allow subsequent brachytherapy using Ru-106 plaque.

## Case report

**Fig. 1:**
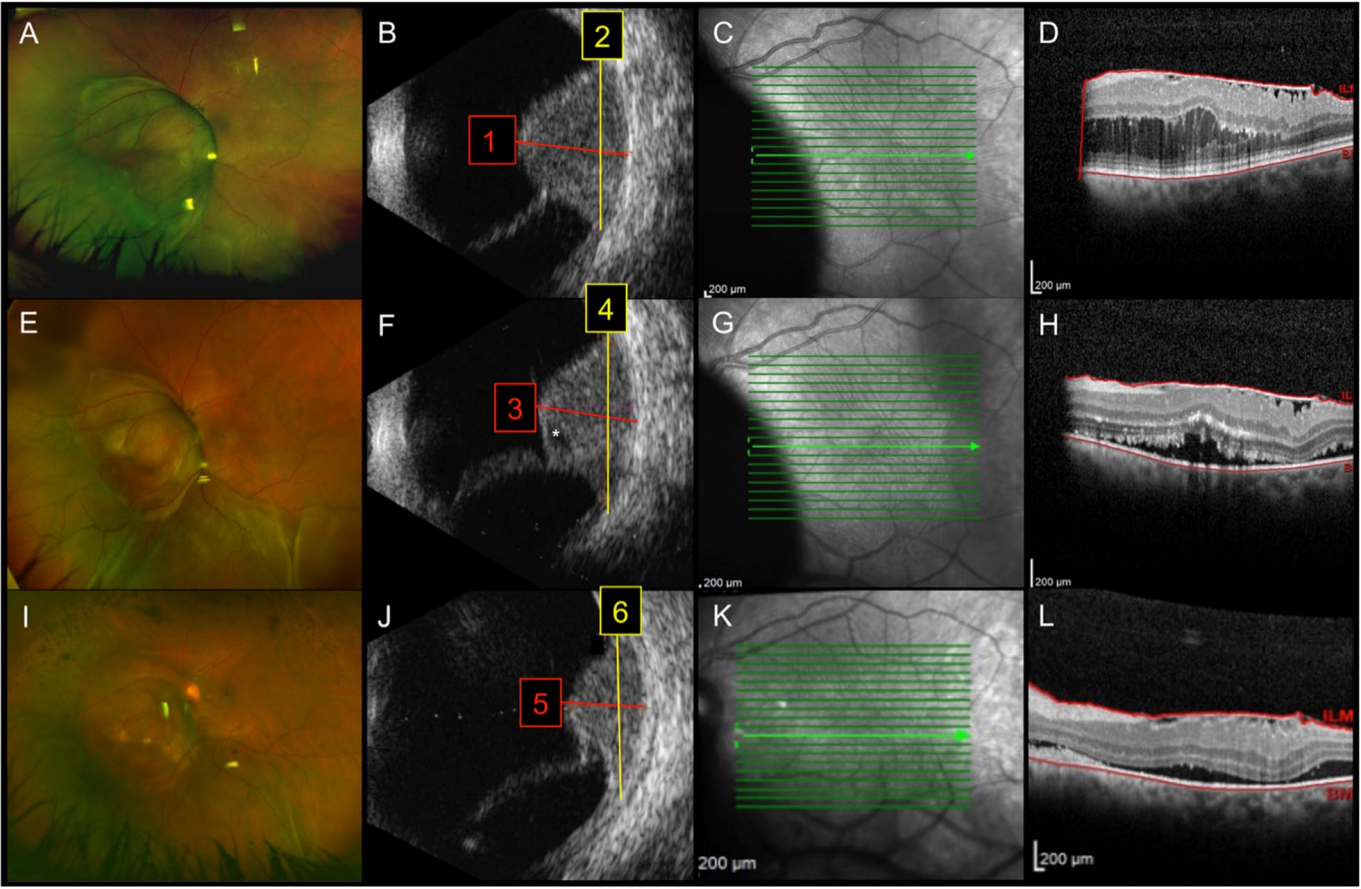
Multimodal assessment of tumor response to intra-arterial chemoreduction. **A**–**D** Baseline, immediately before IAC). Color fundus picture of OS revealed a brownish-pigmented, elevated lesion covering the nasal margin of the optic nerve and extending to the equatorial region also nasally. There was serous retinal detachment over the lesion and around it. **B** Ultrasonography picture showing an elevated choroidal lesion with acoustic hollowness and a thickness of 10.4 mm (1:red line) and a LBD of 13 mm (2:yellow line); the lesion presented superimposing retinal detachment. **C** Near-infrared reflectance image illustrating the shadow of the temporal margin of the choroidal tumor obscuring part of the papillomacular bundle and the temporal margin of the optic disc. **D** Optical coherence tomography showed epiretinal membrane, retinoschisis, and discrete amount of subfoveal fluid. **E**–**H**: three weeks after IAC: **E** Color fundus photo shows partial tumor regression with overlying retinal detachment (mainly inferiorly); **F** On Ultrasonography, choroidal tumor thickness was 7.9 mm (3:red line) and LBD was 11.9 mm (4:yellow line); cystic changes suggestive of necrosis were also verified (asterisk); **G** On infrared reflectance image, there was evident reduction of papillomacular bundle shadowing by the choroidal lesion and optical coherence tomography **H** showed a discrete increase on subfoveal fluid, with maintenance of the remaining retinal architecture. **I**–**L**: 20 weeks after IAC): **I** Most of the optic nerve was now visible on color fundus picture. It also shows further tumor size reduction, which is corroborated by ultrasound measurements (**J**): thickness: 4.9 mm (5:red line), LBD: 8.9 mm (6:yellow line). **K** Infrared reflectance demonstrated complete tumor regression in the papillomacular bundle. **L** OCT illustrates persistent discrete subretinal fluid

A 61-year-old female patient, presented for an ocular oncology consultation complaining of reduced visual acuity and central-temporal scotoma for 1 month in her left eye. Visual acuity was 20/20 in the right eye (OD) and 20/125 in left eye (OS). The anterior segment and intraocular pressure were normal in both eyes, as was fundus examination of the OD. Fundus examination of OS revealed a brownish-pigmented, elevated lesion covering the optic nerve and extending to the equatorial region also nasally. There was serous retinal detachment over the lesion and around it (Fig. 1A, B). The infrared image showed the shadow of the choroidal tumor obscuring part of the papillomacular bundle and the temporal margin of the optic disc (Fig. 1C). Optical coherence tomography showed epiretinal membrane and possible tumor mechanically induced retinoschisis (Fig. 1D). On ultrasonography evaluation, the lesion presented superimposing retinal detachment, acoustic hollowness and had a basal diameter of 13.0 mm and an apical height of 10.4 mm leading to the diagnosis choroidal melanoma (Fig. 1B). Systemic screening was negative for other primary tumors and metastasis. At this point, the patient was offered I-125 plaque (only available at other oncology centers, mainly private practice ones), external beam radiation therapy (EBRT), and enucleation. Since the patient could not afford I-125 brachytherapy, and declined EBRT due to its known side effects, we offered an alternative compassionate use of intra-arterial chemoreduction with Melphalan followed by Ru-106 plaque to avoid enucleation. After extensive discussion of all potential risks and benefits of these procedures, the patient opted to undergo intra-arterial chemotherapy (IAC) with 7.5 mg of Melphalan after selective catheterization of the ophthalmic artery under local anesthesia.

Three weeks after Melphalan IAC, her visual acuity was 20/200 and there was partial regression of the tumor and overlying retinal detachment, as verified on color fundus photo (Fig. 1E) and optical coherence tomography (OCT) (Fig. 1H). On infrared reflectance image, there was evident reduction of papillomacular bundle shadowing (Fig. 1G) and OCT showed discrete subfoveal fluid and regression of retinoschisis (Fig. 1H). On ultrasound evaluation, the largest basal diameter (LBD) was 11.9 mm and thickness was 7.9 mm; cystic changes suggestive of necrosis were also noted. (Fig. 1F) Ru-106 brachytherapy was then performed using a 24-mm notched plaque. Due to tumor thickness and previous IAC, the target dose to the tumor apex was 65 Gy delivered over 82 h. Prior to plaque placement, fine needle aspiration biopsy was performed using binocular indirect ophthalmoscopy to sample the tumor and evaluate cellular viability after IAC. There was limited vitreous hemorrhage over the lesion immediately after the biopsy. Cytology analysis revealed no viable melanocytes; there were only very rare lymphocytes and histiocytes. One week after surgery, the patient presented visual acuity of 20/800 in OS. There was biopsy-related vitreous hemorrhage and an increase of retinal detachment related to radiation treatment. Ten weeks after brachytherapy (13 weeks after IAC), her visual acuity was HM and there was little clearance of the vitreous hemorrhage. On indirect ophthalmoscopy, only the optic nerve and a small portion of the superior peripheral retina could be visualized. On ultrasound evaluation, the LBD was 9.9 mm and thickness was 6.5 mm. There was still an inferior retinal detachment, and the retina seemed more fixed than previous evaluations. Since the tumor thickness was approximately 4 mm smaller (6.5 mm) compared to presentation, we assumed that tumor control had been achieved and we discussed with the patient the possibility of *pars plana* vitrectomy (PPV) for nonclearing vitreous hemorrhage and possible retinal reattachment during surgery. Patient agreed and three-port 23-gauge PPV was performed and vitreous hemorrhage was aspirated. During surgery, the retinal detachment seemed bullous and no tear was found. For this reason, the peroperative decision was to inject 1.0 mg of triamcinolone acetonide into the vitreous cavity without any retinal manipulation. Six weeks after PPV (20 weeks after IAC), her visual acuity was 20/200 and further reduction of tumor dimensions was observed. The largest basal diameter was 8.9 mm and thickness was 4.9 mm at the patient’s last follow-up 17 weeks after Ru-106 brachytherapy. There was still some subretinal fluid on OCT (Fig. 1I–L).

## Discussion

To our knowledge and based on a thorough PUBMED search, this is the first report of IAC using single agent melphalan as a bridge therapy for choroidal melanoma prior to brachytherapy. The rationale for this exploratory therapy comes from the experience of other authors that have reported the use of IAC for uveal melanoma metastatic to the liver and cutaneous melanoma metastatic to the limb. Melphalan was first used as a regional chemotherapeutic agent in 1957 [8] and was subsequently shown to be cytotoxic to melanoma cells in vitro using human cell line (RPMI 8322) [9]. According to Minor et al., melphalan is particularly suited to regional chemotherapy due to its short half-life, its low toxicity to the vascular endothelium and soft tissue and its relatively linear dose–response relationship with respect to cytotoxicity [10]. Besides melphalan, other drugs such as carboplatin and topotecan may also be tried, but there is no evidence that melanoma cells would respond to these drugs.

Systemic toxicity, mainly related to myelosuppression, heralds the systemic use of high doses of Melphalan. For this reason, several successful therapies, including treatment of cutaneous melanoma of the limbs, metastatic to the liver, and ocular tumors such as retinoblastoma involve the regional use of this chemotherapeutical agent. Our patient did not present any systemic side effect. We performed a complete blood count with differential before and after IAC and there were no significant changes. The lack of systemic toxicity we observed in this case may be related to the low dose of 7.5 mg of melphalan used and the short follow-up. This particular dose of melphalan (7.5 mg) was used because it has been reported to be safe in children with retinoblastoma. Despite the obvious difference in weight and body surface of our patient compared to children with retinoblastoma, tumor basal diameter and thickness were comparable with retinoblastomas treated with IAC [11]. Additionally, the dose of Melphalan used for 1 session of IAC offers lower risk of retinal toxicity [12] especially when subsequent brachytherapy was planned. Finally, despite a residual retinal detachment, our patient did not present significant signs of retinal toxicity thus far. In fact, the most remarkable finding was the increase of visual filed our patient experienced. She clearly reported decrease of central and temporal scotomas after IAC. This was probably related to tumor reduction (both diameter and thickness) and serous retinal detachment decrease (Fig. 1E–H). The tumor thickness reduced from 10.4 mm at baseline to 7.9 mm 3 weeks after IAC, when she underwent ruthenium brachytherapy. After brachytherapy, there was further decline of tumor thickness to 4.9 mm. (Fig. 1J).

This exploratory treatment was only considered because Ru-106 plaques (available through the Brazilian public health system) have limitations to tumor dimensions, especially thickness, the patient had no financial means to seek other alternative conservative therapies, and she declined EBRT and enucleation. In addition, one small portion of the tumor was over the optic nerve and ruthenium brachytherapy alone could not treat the entire lesion. Considering these circumstances and after detailed informed consent was obtained, the patient agreed to be treated with combination IAC and subsequent Ru-106 plaque that delivered 65 Gy to tumor apex during a period of 82 h. The therapeutic dose chosen was lower than recommended by the Collaborative Ocular Melanoma Study (85 Gy) to limit the scleral dose and more serious ocular complications. This dose was also based on the equivalent total dose (50–70 Gy) reported in studies utilizing proton beam therapy and stereotactic radiosurgery [2].

This case showed that IAC with Melphalan could lead to a 20% reduction on choroidal melanoma thickness 3 weeks after the procedure. Despite this important reduction, the present report has limitations such as short follow-up and lack of other toxicity evaluations such as ERG. One should bear in mind that this is an early and preliminary report, and that the applicability of melphalan IAC chemoreduction needs to be further verified in larger series with longer follow-up.

## Conclusion

This case illustrates the feasibility of combining induction IAC prior to ruthenium brachytherapy for large choroidal melanoma. Melphalan IAC could lead to a 20% reduction on choroidal melanoma thickness 3 weeks after the procedure and subsequent brachytherapy was associated to additional 37% reduction (around 57% of total thickness reduction) after 20 weeks of initial therapy.

## Data Availability

Data sharing is not applicable to this article as no datasets were generated or analysed during the current study.
